# The Mediating Role of Rumination in the Relationship Between Mindful Awareness and Athlete Burnout

**DOI:** 10.3390/healthcare14121795

**Published:** 2026-06-22

**Authors:** Selin Biçer Baikoğlu, Suzan Dal, Sinan Avcı, Sevim Güllü, Meltem Özağır, Yunus Şahinler, Renas Zaman, Orkun Akkoç

**Affiliations:** 1 Faculty of Sport Science, Istanbul University-Cerrahpaşa, Istanbul 34098, Türkiye; selin.bicerbaikoglu@iuc.edu.tr (S.B.B.); sinan.avci@iuc.edu.tr (S.A.); sevim.gullu@iuc.edu.tr (S.G.); orkun.akkoc@iuc.edu.tr (O.A.); 2 Faculty of Physical Education and Sports Sciences, İstanbul Gelişim Üniversity, Istanbul 34310, Türkiye; mozagir@gelisim.edu.tr (M.Ö.); ysahinler@gelisim.edu.tr (Y.Ş.); 3 Faculty of Movement & Rehabilitation Sciences, KU Leuven, 3000 Leuven, Belgium; renaszamann@gmail.com

**Keywords:** sport, mindfulness, rumination, athlete burnout

## Abstract

Background/Objectives: This study aimed to examine whether rumination statistically accounts for the indirect association between mindfulness and burnout among athletes using a quantitative, cross-sectional, and non-interventional research design. Methods: The sample consisted of 484 licensed athletes (157 females, 32.4%; 327 males, 67.6%) actively involved in team sports, individual sports, and e-sports in Istanbul during the 2024–2025 period. Data were collected using the Athlete Mindfulness Questionnaire, the Sport Competition Rumination Scale, and the Athlete Burnout Questionnaire. Statistical analyses were conducted using IBM SPSS 25 and Hayes’ PROCESS Macro (Model 4), and mediation effects were tested using a bootstrap procedure with 5000 resamples and 95% confidence intervals. Results: The findings revealed significant relationships among mindfulness, rumination, and burnout. Mindfulness was positively associated with rumination, and rumination was significantly associated with athlete burnout. Furthermore, when rumination was included in the model, the direct relationship between mindfulness and burnout became non-significant. Given the cross-sectional nature of the study, the findings should be interpreted as relational rather than causal. Conclusions: In conclusion, the findings suggest that the relationship between mindfulness and burnout may be indirectly shaped through ruminative cognitive processes depending on contextual and cognitive-regulatory factors. The results further suggest that the associations between mindfulness, rumination, and burnout may vary according to competitive context and individual cognitive processing patterns.

## 1. Introduction

Sport is not merely a process of physical performance; rather, it represents a complex experience in which mental resilience, emotional balance, and cognitive flexibility are considered together. In this context, sport can be conceptualized as a multidimensional performance domain shaped by the interaction of physical capacity with cognitive and emotional processes. In contemporary sport settings, performance is increasingly conceptualized as a multidimensional construct shaped not only by physical capacity, but also by cognitive, emotional, and attentional processes. This perspective is also relevant for e-sport athletes, who are exposed to competitive pressure, sustained attentional demands, performance evaluation, and psychological stress similar to those experienced in traditional sports. Therefore, e-sports can also be considered a performance domain within the sport psychology literature, particularly in terms of mental performance and psychological processes. From the preparation phase through competition and the post-competition period, athletes experience and manage a wide range of emotions simultaneously [1,2]. Sustaining athletic performance depends largely on athletes’ ability to cope with various stressors, including intensive training loads, competitive pressure, and performance expectations. Responses to stress may vary depending on its source; however, prolonged exposure to psychological demands may, over time, contribute to the emergence of a syndrome referred to as athlete burnout [3].

Burnout is characterized by emotional, cognitive, and physical exhaustion, accompanied by symptoms such as fear of failure [4,5]. It is generally defined as a state of depletion experienced by individuals who work intensively and invest heavily in their professional roles over extended periods. The concept of burnout was first introduced into the literature by Freudenberger in 1974 [4]. While burnout has been examined across various life domains, its application to sport was first addressed by Thomas D. Raedeke [3]. In the sport context, burnout encompasses a range of experiences, including reduced enjoyment of sport participation, emotional disengagement, and a diminished desire to continue involvement in sport activities that were previously perceived as meaningful and enjoyable [3]. These experiences are commonly associated with factors such as excessive training demands, pressure arising from high performance expectations, conflicts between expectations, and perceived inadequacy in meeting performance-related demands [6]. When such stressors persist without effective resolution, the process may ultimately result in sport dropout [3,7].

Burnout in athletes may manifest through physical, cognitive, and psychological symptoms. Physical symptoms typically involve reduced energy levels, which may indirectly lead to decreased performance. Cognitive symptoms include difficulties in maintaining attention, impaired focus, and reduced concentration. Psychological symptoms may involve motivational difficulties and challenges in sustaining effort and engagement [8]. In this regard, athlete burnout represents a multidimensional process associated with prolonged stress exposure and excessive demands, potentially affecting both psychological functioning and performance levels. It is also considered to be related to athletes’ subjective well-being and psychological resilience [9,10].

One of the key cognitive processes implicated in the development of burnout is rumination [11,12]. Rumination is defined as a repetitive and persistent pattern of thinking in which individuals mentally revisit negative experiences, focusing excessively on mistakes or perceived failures [13]. Rather than engaging in active problem-solving, individuals become passively absorbed in recurring negative thoughts and emotions, resulting in maladaptive cognitive cycles. Such ruminative tendencies may extend beyond constructive reflection, leading to fixation on past events. This process has been associated with increased anxiety, reduced motivation, and intensified burnout symptoms [12,13].

Managing rumination is considered important for athletes’ cognitive and psychological health as well as for sustaining athletic performance. To cope effectively with negative thoughts and emotional states, athletes must first be able to recognize and become aware of their internal experiences. Developing psychological skills that support emotion regulation may facilitate this process. Within this framework, mindfulness has emerged in the literature as a psychological resource with the potential to mitigate the negative effects of rumination.

Kabat-Zinn [14], a leading figure in mindfulness research, defines mindfulness as the ability to intentionally direct attention to present-moment internal and external experiences in a non-judgmental and accepting manner. In sport settings, mindfulness may help athletes maintain focus on present-moment demands and direct attention toward relevant performance cues [15,16,17]. Through mindfulness-based practices, athletes may learn to adapt to and accept their experiences [18], regulate emotions, and maintain mental balance [19]. From a global health perspective, mindfulness can be conceptualized as a complementary and integrative psychological approach, particularly in the context of preventive mental health.

Individuals with higher levels of mindfulness tend to respond less reactively to negative thoughts, demonstrate stronger coping skills in the face of stress, and exhibit greater emotional stability [20]. Research in sport psychology indicates that athletes with higher mindfulness levels are better able to sustain concentration, manage competitive anxiety and stress, and generally report lower levels of burnout [21,22].

However, the existing literature also suggests that mindfulness does not exert uniform effects across different athlete populations or contextual conditions [21,23]. Although the overall evidence is generally favorable, recent umbrella review findings have highlighted substantial methodological heterogeneity across mindfulness-based intervention studies, indicating that conclusions regarding effectiveness should be interpreted cautiously [23]. In other words, even when individuals exhibit high levels of mindfulness, persistent focus on past mistakes or negative performance-related scenarios, combined with contextual factors that limit present-moment engagement, may constrain the psychological benefits typically associated with mindfulness. This indicates that mindfulness may not automatically reduce ruminative thinking in all contexts. Accordingly, considering rumination as a cognitive-relational factor associated with mindfulness and burnout appears theoretically meaningful. Recent evidence has further demonstrated that mindfulness and cognitive emotion regulation strategies may jointly explain the association between psychological stress and athlete burnout, suggesting that cognitive processes play a central role in burnout development [24].

Empirical studies examining the indirect associations among mindfulness, rumination, and athlete burnout remain limited. While mindfulness has been associated with athlete burnout, this association may be indirectly related to ruminative thought processes. Based on this perspective, rumination may represent a statistically meaningful indirect relational component in the association between mindfulness and burnout within a cross-sectional framework.

**Figure 1:**
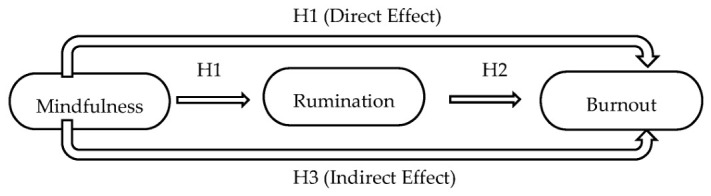
Cross-sectional indirect association model.

Against this background, the aim of the present study is to examine the mediating role of rumination in the relationship between mindfulness and burnout in athletes, and to explore the cognitive processes through which mindfulness is associated with athlete burnout. Within this framework, the relationships among mindfulness, rumination, and burnout are examined using a mediation model, without implying causal inference. Accordingly, examining mindfulness within an integrative health framework may provide important insights into psychological well-being and mental health in athletes (Figure 1).

## 2. Method

### 2.1. Research Design

This study was conducted using a quantitative research approach based on a relational survey model to examine the relationships among mindfulness, rumination, and burnout in athletes. The study employed a cross-sectional and non-interventional design. The relationships among variables and the statistical mediating role of rumination were evaluated through mediation analysis, without making causal inferences. Given the cross-sectional and non-interventional nature of the study, the mediation analysis conducted in the present research should not be interpreted as evidence of causal mediation or temporal sequencing. Rather, the findings reflect statistically significant indirect associations among the variables measured at a single point in time.

### 2.2. Procedure

This study consisted solely of a survey design and collected anonymous and confidential data from participants who voluntarily agreed to complete the questionnaire. All procedures were conducted in strict accordance with the ethical principles outlined in the Declaration of Helsinki. Ethical approval was obtained from the Social and Human Sciences Ethics Committee of Istanbul Aydın University (Approval No: 2025/3, dated 20 March 2025). Data collection commenced after obtaining ethical approval and the necessary permissions for the use of the relevant measurement scales. Data were collected over a period of approximately 4–6 months through face-to-face administration, and voluntary informed consent forms were obtained from an initial sample of 485 athletes.

Participants were recruited from university sport teams, amateur sport clubs and e-sport communities located in Istanbul. Recruitment was conducted through direct communication with coaches, team administrators, and club representatives. Athletes who met the eligibility criteria and voluntarily agreed to participate were invited to complete the questionnaires during training sessions or organized team meetings.

Prior to questionnaire administration, participants were informed in detail about the purpose of the study, and it was explicitly emphasized that participation was entirely voluntary. Participants were also clearly informed that the collected data would be used solely for scientific purposes, would not be shared with any individual or institution other than the researchers, and would be handled in accordance with confidentiality principles.

Completion of the questionnaires took approximately 20–30 min per participant, and all procedures were conducted under similar conditions.

### 2.3. Participants

The sample of the study consisted of athletes residing in Istanbul during the 2024–2025 period who were licensed and actively competing within a sports club in team sports, individual sports, or e-sports, and who voluntarily agreed to participate in the study. The research group was formed using a convenience sampling method from among athletes who met the predefined inclusion criteria. Initially, 485 athletes participated in the study. However, one participant was excluded from the final analyses due to incomplete demographic information. Therefore, the final sample consisted of 484 athletes. Although the sample included a higher proportion of male athletes, this distribution reflects the accessibility of active athletes during the data collection process. Therefore, potential gender-related differences should be interpreted cautiously, and future studies are encouraged to examine these relationships using more balanced gender distributions.

Convenience sampling was preferred in the present study due to practical limitations related to accessing active athletes from different sport disciplines within a limited data collection period. Probability-based sampling was not feasible because of time constraints, accessibility limitations, and institutional permissions associated with sports clubs and organizations. Therefore, convenience sampling was considered an appropriate and commonly used strategy in sport psychology research involving active athlete populations. Although this approach may limit the generalizability of the findings, it enabled the inclusion of athletes from different sport branches and competitive contexts, thereby increasing sample diversity. This broader sampling strategy was preferred to capture a wider range of competitive cognitive experiences associated with athlete burnout and rumination processes. Because recruitment was conducted through voluntary participation within accessible athlete groups, an exact response rate could not be calculated.

Of the participants, 37.2% were team sport athletes, 31.8% were individual sport athletes, and 31.0% were e-sport athletes. Participants’ ages ranged between 18 and 34 years. The sample comprised 157 female athletes (32.4%) and 327 male athletes (67.6%). Athletes represented a variety of team and individual sport disciplines.

In this study, team sport athletes, individual sport athletes, and e-sport athletes were considered together in order to enhance sample diversity. Although athletes from different sport categories were included to enhance sample diversity, these groups may differ in terms of performance demands, training structure, competitive stressors, and psychological characteristics. Therefore, combining heterogeneous athlete groups within a single analytical model may limit the specificity of the findings. E-sport athletes were included in the study because, similar to traditional athletes, they are exposed to competitive pressure, performance expectations, and cognitively demanding environments that may contribute to rumination and burnout processes. Therefore, e-sports were considered relevant to the scope of the present study, which focuses on the cognitive and psychological dimensions of athlete burnout. Sport-type-based comparative analyses were beyond the scope of the present study. This approach is considered a limitation when evaluating the generalizability of the findings.

Inclusion criteria were defined as being an active athlete for at least one year, engaging in regular training in the relevant sport discipline, voluntarily agreeing to participate in the study, and completing the data collection instruments in full. Exclusion criteria included not being an active athlete, not engaging in regular training, incomplete or incorrect completion of the questionnaires, and lack of voluntary participation.

### 2.4. Data Collection Instruments

#### 2.4.1. Demographic Information Form

Information regarding participants’ age, gender, sport discipline, and years of sport experience was collected using a demographic information form.

#### 2.4.2. Athlete Burnout Questionnaire

The Athlete Burnout Questionnaire (ABQ) was developed by Raedeke and Smith [3] and adapted to the Turkish athlete population by Kelecek [25]. The Turkish version of the scale consists of 13 items and includes three subdimensions: reduced sense of accomplishment, physical and emotional exhaustion, and sport devaluation. Items are rated on a 5-point Likert scale ranging from 1 (Strongly disagree) to 5 (Strongly agree). Subscale and total scores are calculated by averaging the relevant items. Higher scores indicate higher levels of athlete burnout. In the present study, the internal consistency coefficient of the scale was Cronbach’s alpha ≈ 0.87.

#### 2.4.3. Athlete Mindfulness Questionnaire

The original version of the Athlete Mindfulness Scale was developed by Thienot et al. [16] and adapted to the Turkish athlete population by Emre Ozan Tingaz [26]. The scale consists of 15 items across three subdimensions: present-moment awareness, non-judgmental acceptance, and acceptance. Items are rated on a 5-point Likert scale ranging from 1 (Never) to 5 (Always). Higher scores reflect higher levels of athlete mindfulness. In this study, the overall internal consistency coefficient of the scale was Cronbach’s alpha ≈ 0.84.

#### 2.4.4. Sport Competition Rumination Scale

The Sport Competition Rumination Scale is the Turkish adaptation of the scale originally developed by Michel-Kröhler et al. [27] and was adapted to the Turkish athlete sample by Karafil and Pehlivan [28]. The scale consists of 6 items with a single-factor structure designed to assess repetitive and involuntary ruminative thoughts experienced after sport competitions. Higher total scores indicate greater levels of ruminative thinking. In the Turkish adaptation study, the internal consistency coefficient was reported as Cronbach’s alpha = 0.91. In the present study, the internal consistency coefficient of the rumination scale was found to be Cronbach’s alpha = 0.89.

#### 2.4.5. Data Analysis

Data were analyzed using IBM SPSS Statistics 25.0 software. Prior to analysis, the dataset was examined for missing data, outliers, and assumptions of normality. Descriptive statistics, including frequencies (*n*) and percentages (%), were calculated to summarize participants’ demographic characteristics and are presented in Table 1.

The assumption of normality required for parametric analyses was evaluated using skewness and kurtosis values, as well as Normal Q–Q Plot graphs. Skewness and kurtosis values for the rumination, mindfulness, and burnout variables were found to fall within the range of −1.5 to +1.5, and the Q–Q plots indicated that observed values were aligned with the theoretical normal distribution line. Based on these findings, the data were considered sufficiently normally distributed, and parametric statistical analyses were applied. In addition to regression coefficients, effect size indicators (Cohen’s f^2^) were calculated to evaluate the practical magnitude of the observed relationships.

To examine the relationships among the study variables, linear regression analyses were conducted. In the first step, the association between mindfulness and rumination was tested; in the second step, the associations of mindfulness and rumination with burnout were examined simultaneously. Standardized (β) and unstandardized (b) coefficients, t-values, significance levels (*p*), and explained variance (R^2^) were reported. Multicollinearity assumptions were evaluated using tolerance and Variance Inflation Factor (VIF) values.

To examine the mediating role of rumination in the relationship between mindfulness and burnout, a mediation analysis was conducted using Hayes’ PROCESS Macro Model 4. The significance of indirect effects was tested using the bootstrap method with 5000 resamples, and 95% confidence intervals were calculated. Confidence intervals that did not include zero were interpreted as indicating statistically significant indirect effects. For all statistical analyses, the level of significance was set at *p* < 0.05. Internal consistency coefficients for all scales used in the present sample were examined using Cronbach’s alpha values and were found to be within acceptable ranges. Prior to analysis, the dataset was screened for missing values and outliers. Cases with incomplete responses were excluded from the analyses. Assumptions related to normality and multicollinearity were also evaluated before conducting regression analyses. Questionnaires containing substantial missing data, inconsistent response patterns, or clearly invalid markings were excluded prior to the final analysis in order to improve data quality and analytical reliability. During the data screening process, no missing item-level data were detected in the final dataset. However, questionnaires containing incomplete demographic information, repetitive response patterns (e.g., identical responses across all items), or logically inconsistent markings were considered invalid and excluded prior to analysis. Univariate outliers were examined using standardized z-scores, and no extreme values exceeding ±3.29 were identified. A post hoc statistical power analysis was conducted using G*Power 3.1 software to assess sample adequacy. Based on a medium effect size (f^2^ = 0.15), an alpha level of 0.05, and two predictors included in the regression model, the achieved statistical power exceeded 0.95 for the final sample (*n* = 484). This result indicates that the sample size was sufficient for detecting statistically meaningful effects.

## 3. Results

Table 1 shows the gender distribution of participants: 67.6% are male (*n* = 327) and 32.4% are female (*n* = 157). This finding indicates that male participants are more numerous in the sample. Regarding age groups, the vast majority of participants are in the 18–24 age range (69.6%; *n* = 337). This is followed by the 25–29 age group (19.7%; *n* = 95) and the 30 years and older group (10.7%; *n* = 52). This distribution reveals that the sample is predominantly composed of young adults. When the age of participation in sports was examined, the 1–3 years (29.1%; *n* = 141) and 10 years and over (29.5%; *n* = 143) groups stood out, while the sample had a balanced distribution in terms of sports experience. The 4–6 years (26.7%; *n* = 129) and 7–9 years (14.7%; *n* = 71) groups also held significant positions. When the distribution according to athlete category was examined, it was determined that 37.2% of the participants nts were team athletes, 31.8% were individual athletes, and 31.0% were e-sports players.

Examining the skewness and kurtosis values for the rumination, mindful awareness, and burnout variables in Table 2, it is observed that all values fall within the range of −1.5 to +1.5. This indicates that the distributions of the variables are sufficiently close to a normal distribution.

The skewness (−0.330) and kurtosis (−0.185) values of the rumination variable indicate that the distribution is quite close to normal and within acceptable limits. Similarly, the skewness (−0.403) and kurtosis (0.187) values of the mindfulness variable also point to a symmetrical distribution. The skewness (0.308) and kurtosis (−0.680) values of the burnout variable show that although the distribution is slightly skewed to the right, it does not violate the assumption of normal distribution.

Table 3 presents the descriptive statistics and zero-order correlations among mindfulness, rumination, and burnout variables. The findings indicate that participants reported moderate levels of mindfulness (M = 3.42, SD = 0.64), rumination (M = 2.87, SD = 0.71), and burnout (M = 2.95, SD = 0.68). Correlation analyses revealed a statistically significant positive association between mindfulness and rumination (r = 0.52, *p* < 0.001). This finding suggests that higher levels of mindfulness were associated with higher levels of ruminative thinking within the present sample. In contrast, the relationship between mindfulness and burnout was weak and non-significant (r = 0.09), indicating that mindfulness was not directly associated with burnout at the bivariate level. Additionally, rumination demonstrated a significant positive relationship with burnout (r = 0.21, *p* < 0.001), suggesting that increased ruminative thinking was associated with higher burnout levels among athletes.

Table 4 shows that the regression analysis results indicate that mindful awareness is a strong and statistically significant predictor of the dependent variable. The regression coefficient for mindfulness (b = 0.21, SE = 0.02) was found to be significant (t = 13.46, *p* < 0.001), indicating a high contribution of this variable to the model. The standardized regression coefficient (β = 0.52) reveals that a one-unit increase in mindfulness level is associated with a moderately high increase in the dependent variable. The effect size for the mindfulness → rumination model was large (f^2^ = 0.37), indicating a substantial contribution of mindfulness to the prediction of rumination.

The regression analysis results presented in Table 5 reveal the combined effect of mindfulness and rumination on burnout. The model is generally significant (R^2^ = 0.06, F (2, 481) = 14.36, *p* < 0.001). This finding indicates that the independent variables explain approximately 6% of the variance in burnout. When the findings are examined, it is determined that rumination significantly and positively predicts burnout (b = 0.03, SE = 0.01, t = 4.02, *p* < 0.001, β = 0.21). This result shows that as the level of rumination increases, the level of burnout also increases significantly. In contrast, the direct effect of mindfulness on burnout is insignificant (b = 0.003, *p* = 0.358, β = 0.05). This suggests that when rumination is included in the model, the effect of conscious awareness on burnout disappears, and the effect occurs indirectly. The overall effect size for the burnout model was small (f^2^ = 0.06), suggesting a limited but statistically meaningful explanatory contribution of the predictors.

**Figure 2:**
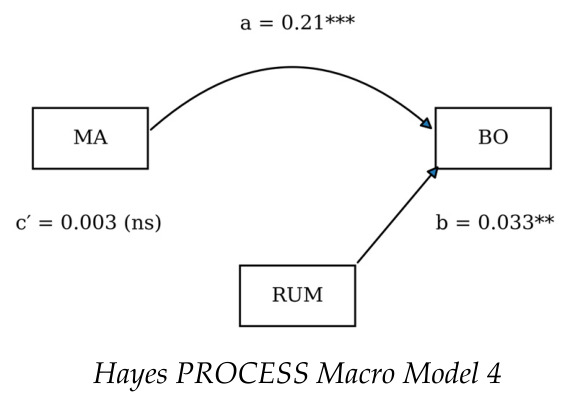
Analysis results show that mindful awareness (MA) was significantly and positively associated with rumination (RUM) (*a* = 0.21, *p* < 0.001). Rumination, in turn, was found to be significantly associated with burnout (BO) (*b* = 0.03, *p* < 0.01). However, when rumination was included in the model, the direct effect of mindful awareness on burnout became insignificant (*c′* = 0.003, *p* > 0.05). Indirect effects analysis using the bootstrap method revealed that the effect of mindful awareness on burnout occurs indirectly through rumination, and this indirect effect is statistically significant. These findings suggest a statistically significant indirect association between mindful awareness and burnout through rumination. Note. *** *p* < 0.001; ** *p* < 0.01; ns = non-significant.

Table 6 presents the bootstrap indirect effect analysis examining the association between mindfulness and burnout through rumination. The indirect effect was statistically significant (ab = 0.007, Boot SE = 0.002), and the 95% bootstrap confidence interval did not include zero (95% Boot LLCI = 0.0034, 95% Boot ULCI = 0.0107). These findings indicate a statistically significant indirect cross-sectional association between mindfulness and burnout through rumination (Figure 2).

## 4. Discussion and Conclusions

In the present study, the mediating role of rumination in the relationship between mindfulness and burnout among athletes was examined, and the findings indicated that rumination appears to represent a statistically significant indirect cross-sectional association linking mindfulness and burnout. These results suggest that the association between mindfulness and burnout should not be interpreted as a direct effect; rather, it appears to be shaped indirectly through ruminative cognitive processes. In particular, the analyses presented in Table 3 indicated that mindfulness was significantly and positively associated with rumination, and that rumination was significantly associated with burnout. The loss of statistical significance in the direct relationship between mindfulness and burnout after the inclusion of rumination in the model indicates that rumination may represent a statistically meaningful indirect cross-sectional association within this relationship. Due to the cross-sectional design of the study, this finding should be interpreted as a pattern of indirect associations rather than as evidence of causality.

Previous research has emphasized mindfulness as a potential stress-buffering resource in sport contexts. Recent evidence has further demonstrated that mindfulness is positively associated with mental resilience in athletes, suggesting that mindful athletes may be better equipped to cope with competitive stressors and maintain psychological well-being [9]. Similarly, Birrer et al. [29] suggested that mindfulness-based interventions may support athletes’ psychological skills and performance continuity through the regulation of rumination, negative effects, and stress, thereby providing theoretical support for the framework of the present study.

However, Josefsson et al. [30] emphasized that the capacity of mindfulness to reduce rumination may not be sufficient under all conditions and should be considered in conjunction with emotion regulation skills to achieve effective outcomes. In the absence of such regulatory capacities, athletes may become more focused on performance errors and internal cognitive content. This perspective highlights the need to interpret the direction of the mindfulness–rumination relationship in a context-sensitive manner, consistent with the findings of the present study.

Contrary evidence has also been reported in the literature. Britton [31] argued that in highly competitive sport environments characterized by continuous performance evaluation, mindfulness practices may increase rumination and emotional hyper-awareness. From this perspective, mindfulness does not necessarily assume an automatically regulatory role for athletes; instead, it may create conditions under which ruminative thoughts become more salient and persistent. This interpretation offers a contextual explanation for the findings independent of potential measurement-related issues.

Building on this perspective, the positive association observed between mindfulness and rumination should be interpreted cautiously. Although mindfulness is traditionally conceptualized as a protective factor against repetitive negative thinking, emerging evidence suggests that its effects may vary across contexts and populations. Alternative explanations related to measurement interpretation, self-focused attention, and cultural factors should also be considered. Therefore, this finding should be regarded as exploratory and interpreted within the contextual limitations of the present study.

The finding that rumination was significantly associated with burnout (Table 4) is strongly supported by the existing literature. Rumination has consistently been conceptualized as a maladaptive cognitive process that prolongs stress responses and interferes with effective emotional regulation [13]. In sport settings, persistent ruminative thinking has also been associated with increased performance anxiety, attentional disruption, and reduced psychological recovery, all of which may contribute to burnout symptoms.

The explanatory power of the model was relatively modest, with approximately 6% of the variance in burnout explained by mindfulness and rumination. This finding is not unexpected given the multidimensional nature of athlete burnout, which is influenced by numerous psychological, emotional, environmental, physiological, and sport-specific factors. Therefore, mindfulness and rumination should be interpreted as specific cognitive-relational variables within a broader biopsychosocial framework rather than as comprehensive explanatory mechanisms.

In contrast, the non-significant direct effect of mindfulness on burnout (Table 4) suggests that the potential protective association of mindfulness may not operate in a direct and uniform manner. While a substantial body of the literature has demonstrated a negative association between mindfulness and burnout, emerging evidence indicates that this relationship may be indirect and mediated by cognitive and emotional processes. For instance, Josefsson et al. [30] and McMillen [11] highlight that the effects of mindfulness on burnout are often transmitted through indirect relational patterns such as rumination and emotion regulation. Accordingly, the present findings suggest that mindfulness may be indirectly associated with burnout as part of a broader cognitive system in which these relationships appear to depend on how individuals process and regulate their internal experiences.

The indirect effect findings (Table 5) further clarify this pattern by demonstrating that rumination constitutes a statistically significant cross-sectional association linking mindfulness and burnout. Although the statistical model was significant, the explained variance for burnout remained relatively modest. This finding suggests that athlete burnout is likely influenced by multiple psychological, environmental, physiological, and sport-contextual factors beyond mindfulness and rumination. Therefore, the present findings should be interpreted primarily as indicating statistically meaningful relational patterns rather than a highly predictive explanatory model. More specifically, the results suggest that the relationship between mindfulness and burnout appears to be partially associated with ruminative cognitive processes rather than a direct association. Although the indirect effect was statistically significant, its magnitude was relatively small. Therefore, the observed mediation effect should be interpreted cautiously in terms of practical significance. The findings primarily indicate the presence of a statistically meaningful indirect cross-sectional association rather than a strong practical impact. In this sense, the hypothesized mediating role of rumination is supported at the level of statistical indirect effects; however, given the cross-sectional nature of the data, this pattern should be interpreted as a relational configuration rather than a causal mediation process. These findings therefore suggest that mindfulness, rumination, and burnout operate within an interconnected cognitive framework, where the association between mindfulness and burnout may be influenced by ruminative processing tendencies.

Although the indirect effect was statistically significant, the explanatory power of the model was relatively modest. The current findings suggest that rumination may represent only one of several psychological indirect relational patterns associated with athlete burnout. Athlete burnout is a multidimensional construct influenced by cognitive, emotional, environmental, physiological, and sport-specific factors. Therefore, the present findings should be interpreted cautiously and within the broader context of multifactorial burnout models. In this respect, future research should adopt more comprehensive models including contextual and sport-specific variables associated with athlete burnout.

Additionally, another important limitation of the present study is that several variables associated with athlete burnout were not included in the model. Factors such as training load, injury history, coach-related pressure, perfectionism, sleep quality, social support, competitive anxiety, competitive level, and type of sport may substantially influence burnout symptoms. The exclusion of these variables may partially explain the low explained variance observed in the model. Future studies should incorporate broader biopsychosocial and sport-contextual variables to develop more comprehensive explanatory frameworks. Another important limitation of the present study is that several demographic, sport-related, and psychosocial variables associated with athlete burnout were not included as covariates in the analytical model. Variables such as age, gender, sport experience, athlete category, competitive level, training load, injury history, sleep quality, perfectionism, anxiety symptoms, and coach-related pressure may substantially influence burnout processes. However, the primary aim of the present study was to examine the general cross-sectional relational associations among mindfulness, rumination, and burnout within a parsimonious mediation framework rather than to construct a fully adjusted predictive model. Therefore, these variables were not simultaneously incorporated into the analysis. Future studies are encouraged to test more comprehensive multivariate models, including contextual and sport-specific covariates, in order to improve explanatory power and clarify potential confounding effects.

Although rumination appears to be associated with burnout, practical implications should be interpreted cautiously. Future longitudinal and experimental studies are needed to determine whether reducing rumination may effectively contribute to the prevention of athlete burnout.

One of the most notable findings of the present study was the positive association between mindfulness and rumination, which contrasts with much of the existing literature. This unexpected pattern suggests that the relationship between mindfulness and rumination in athletes may be more context-dependent and complex than traditionally assumed. Factors such as competitive pressure, cultural context, emotion regulation capacities, and possible measurement-related interpretations may have contributed to this finding. Therefore, future longitudinal, experimental, and cross-cultural studies are needed to better understand the conditions under which mindfulness may be associated with increased ruminative tendencies.

Furthermore, it is important to interpret these findings within a cultural context. In Turkish sport culture, and more broadly in collectivist societies, performance evaluation and fear of making mistakes are often highly internalized. Cultural characteristics associated with collectivist social structures and performance-oriented sport environments may represent one possible contextual interpretation of the findings. However, because cultural variables were not directly measured in the present study, these interpretations remain speculative and should be evaluated cautiously. Another limitation of the present study is that the sample consisted only of athletes residing in Istanbul. Therefore, the findings may not fully represent athletes from different regions or competitive environments, and caution should be exercised when generalizing the results. Future studies including more diverse samples may improve the generalizability of the findings.

Age, gender, sport experience, and athlete category were not included as control variables in the model. Future studies may benefit from incorporating these variables into more comprehensive analytical frameworks. Therefore, the findings should be interpreted cautiously, and future studies are encouraged to examine these athlete groups separately. Additionally, homogeneity analyses across sport categories were not conducted. Since team sport athletes, individual athletes, and e-sport athletes may differ in psychological and competitive characteristics, future research should investigate potential group-specific patterns. The competitive level of the athletes (e.g., amateur, semi-professional, or professional) was not included as a control variable in the analysis. Since competitive levels may influence both burnout and cognitive processes, future studies should consider incorporating this variable into the analytical framework. Because all variables were measured using self-report instruments, the findings may be influenced by common method bias and shared response tendencies. Because the present study employed a cross-sectional design, the findings should not be interpreted as evidence of causal mediation or temporal directionality. The observed indirect associations merely reflect statistical relationships among mindfulness, rumination, and burnout measured simultaneously. Longitudinal and experimental studies are required to clarify potential temporal and causal processes underlying these associations. Another limitation of the present study is that mindfulness was evaluated using a total score approach, and mindfulness subdimensions were not analyzed separately. Since different mindfulness components may demonstrate distinct relationships with rumination and burnout, future studies should examine subdimension-specific patterns using more detailed analytical frameworks. Although heterogeneous athlete groups were analyzed within a unified analytical framework, it is possible that the observed relationships may differ across sport categories. Team sport athletes, individual athletes, and e-sport athletes may vary in terms of competitive structure, emotional regulation demands, social interaction patterns, and performance-related stress exposure. Because subgroup analyses and covariate-based comparisons were beyond the primary scope of the present study, potential group-specific differences could not be fully examined. Future studies are encouraged to conduct subgroup-based or multigroup analytical models in order to clarify possible sport-category-specific cognitive patterns.

## Figures and Tables

**Table 1:** Demographic Characteristics of Participants.

Gender		Frequency (*n*)	Percentage (%)
	Female	157	32.4%
	Male	327	67.6%
Age Group	18–24 Years	337	69.6%
	25–29 Years	95	19.7%
	30 Years And Older	52	10.7%
Sports Experience	1–3 Years	141	29.1%
	4–6 Years	129	26.7%
	7–9 Years	71	14.7%
	10 Years And Older	143	29.5%
Athlete Category	Team Athlete	180	37.2%
	İndividual Athlete	154	31.8%
	E-Athlete	150	31.0%

**Table 2:** Skewness and Kurtosis Values of the Variables Used in the Study.

Variable	Skewness	Kurtosis
Rumination	−0.330	−0.185
Mindfulness	−0.403	0.187
Burnout	0.308	−0.680

**Table 3:** Descriptive Statistics and Correlations Among Variables.

Variable	M	SD	1	2	3
1. Mindfulness	3.42	0.64	—		
2. Rumination	2.87	0.71	0.52 **	—	
3. Burnout	2.95	0.68	0.09	0.21 **	—

Note. ** *p* < 0.001.

**Table 4:** The Effect of Mindful Awareness on Rumination.

Variable	b	SE	t	*p*	β
Constant	7.84	1.03	7.61	<0.001	—
Mindfulness	0.21	0.02	13.46	<0.001	0.52

Notes. R^2^ = 0.27, F(1, 482) = 181.24, *p* < 0.001.

**Table 5:** The Effect of Mindfulness and Rumination on Burnout.

Variable	b	SE	t	*p*	β
Constant	1.81	0.20	9.25	<0.001	—
Mindfulness	0.003	0.003	0.92	0.358	0.05
Rumination	0.03	0.01	4.02	<0.001	0.21

Notes. R^2^ = 0.06; F(2, 481) = 14.36; *p* < 0.001.

**Table 6:** Bootstrap Indirect Effect of Mindfulness on Burnout Through Rumination.

Path	Indirect Effect (ab)	Boot SE	95% Boot LLCI	95% Boot ULCI
Mindfulness → Rumination → Burnout	0.007	0.002	0.0034	0.0107

Note. Bootstrap confidence intervals were generated using 5000 resamples.

## Data Availability

The datasets generated and analyzed during the current study are not publicly available due to ethical and participant confidentiality considerations. However, anonymized data may be made available by the corresponding author upon reasonable request and subject to approval by the relevant ethics and institutional regulations.
